# Fermented rice bran extract delays skin aging by increasing the synthesis of collagen and elastin

**DOI:** 10.3389/fphar.2025.1692491

**Published:** 2025-11-17

**Authors:** Xin Yan, Meiqing Yang, Xuyi Cai, Yiwei Shen, Ruihan Jiang, Rizhong Huang, Huai-Kai Shi, Gregory Cheng, Yiwei Wang, Qian Tan, Yuen Yee Cheng, Nannan Xue

**Affiliations:** 1 Department of Burns and Plastic Surgery, Nanjing Drum Tower Hospital, Affiliated Hospital of Medical School, Nanjing University, Nanjing, China; 2 School of Pharmacy, Nanjing University of Chinese Medicine, Nanjing, China; 3 State Key Laboratory of Natural Medicines, China Pharmaceutical University, Nanjing, China; 4 School of Traditional Chinese Medicine, Beijing University of Chinese Medicine, Beijing, China; 5 Asbestos and Dust Diseases Research Institute, Concord, NSW, Australia; 6 Synertechcare Australia Pty Ltd., Sydney, NSW, Australia; 7 Department of Burns and Plastic Surgery, Nanjing Drum Tower Hospital, Nanjing University of Chinese Medicine, Nanjing, China; 8 Institute for Biomedical Materials and Devices (IBMD), Faculty of Science, The University of Technology Sydney, Sydney, NSW, Australia; 9 School of Integrative Medicine, Nanjing University of Chinese Medicine, Nanjing, China

**Keywords:** rice bran extract, UPLC-QTOF-MS/MS, 3D MSF spheroids, elastin, collagen deposition, transepidermal water loss

## Abstract

**Background:**

Rice bran is the outer layer of rice grains (*Oryza sativa*). Due to its rich bioactive components, it has long been used in cosmetics. However, the mechanism by which it delays skin aging remains unclear.

**Methods:**

In this study, volatile polar solvents combined with microbial fermentation were utilized to enhance the yield and bioavailability of functional components in rice bran extract (RBE). The crude RBE was fermented with *Aspergillus oryzae* for 14 days to promote enzymatic decomposition into smaller and more bioavailable molecules. The components in the fermented RBE were qualitatively analyzed by UPLC-QTOF-MS/MS. The expression of collagen in two-dimensional and three-dimensional cell cultures was evaluated by qPCR technology. The expressions of collagen and elastin and the changes in water content and elastic modulus in the skin of mice were evaluated by histopathology, immunofluorescence staining, and transepidermal water loss (TEWL).

**Results:**

Through UPLC-QTOF-MS/MS analysis, eight key compounds, including azelaic acid, ferulic acid, γ-tocotrienols, and squalene, were identified in RBE, mainly lipids and polyphenols. The treatment of RBE significantly upregulated the expression of type I collagen in MSF cells and the expression of type III collagen in MSF 3D cell spheres (by approximately 12 times). The results of tissue staining showed that the content of collagen in the skin after RBE treatment increased by 10% compared with the control group. The results of immunofluorescence staining confirmed that RBE could increase the content of elastin in the skin. The TEWL results showed that the skin moisture content and elastic coefficient of mice treated with RBE increased by more than 10% compared with those of the untreated group.

**Conclusion:**

Both *in vitro* and *in vivo* studies have shown that RBE can significantly improve the synthesis of collagen and elastin in the skin, reduce water loss in mouse skin, increase collagen deposition in the skin, and ultimately improve skin elasticity and overall quality. This green, solvent-efficient, and fermentation-enhanced approach offers a sustainable strategy for utilizing rice bran as a high-value cosmetic ingredient with strong potential for skincare applications.

## 1 Introduction

Rice bran extract (RBE), derived from the outer layer of *Oryza sativa* grains, is well-known for its rich source of bioactive compounds, including γ-oryzanol, tocopherols, tocotrienols, ferulic acid, phytosterols, and essential fatty acids (Perez-Ternero et al., 2017). In the past decades, rice bran has gained increasing attention in dermatology due to its great potential in antioxidant, anti-inflammatory, and photoprotective properties (Lai et al., 2019; Wang M. L. et al., 2025). Recent investigations have revealed that RBE components can modulate skin barrier function, reduce transepidermal water loss (TEWL), and improve skin hydration and smoothness (Ha et al., 2018; Wang M. L. et al., 2025). Antioxidant-rich RBE products have also been reported to play a critical role in neutralizing the reactive oxygen species (ROS) generated by UV exposure and pollutants, thereby reducing oxidative stress-induced skin aging (Linsaenkart et al., 2023). In addition, fermented RBE has been shown to inhibit melanogenesis and suppress pro-inflammatory cytokines, showing that RBE is a promising candidate for the management of hyperpigmentation and inflammatory dermatoses (Chung et al., 2009; Yu et al., 2019). Furthermore, RBE exhibits moisturizing properties by reinforcing the lipid matrix of the stratum corneum, which may help in treating xerosis and sensitive skin conditions. Its biocompatibility and non-irritating nature make it suitable for incorporation into cosmetic and dermatological formulations, including creams, serums, and sunscreens.

Due to its broad therapeutic potential, rice bran oil is used in many clinical applications, such as sunscreens and topical anti-aging products (Bernardi et al., 2011). Moreover, various methods of extracting bioactive compounds from *Oryza sativa* have been previously reported (Lu et al., 2025). However, given the diversity in molecular structures and associated solubility profiles among these bioactive compounds, the extraction techniques inevitably recover only a selection of what may be present in a harvested plant. Crude rice bran oil is known to contain numerous bioactive compounds, only some of which are saponifiable. Therefore, it is considered that rice bran oil extracted using traditional techniques fails to include a large amount of unsaponifiable matter that could exert useful physiological effects on the human body. Additionally, routine extraction methods used on *Oryza sativa* include solvents or mixtures of solvents, such as acetone, acetic acid, and acidified methanol, which can be difficult to remove from the extract and can be potentially harmful to human cells. Furthermore, routine extraction methods often utilize harsh conditions, including high temperatures, which can further denature or degrade bioactive compounds into a less effective or inactive form.

As one of the important methods of rice bran processing, fermentation products can be used as raw materials for high-quality feed and food production. Fermentation is an important method of rice bran processing and can be used to produce high-quality feed and food (Yu et al., 2019). By accurately controlling the fermentation process parameters, the active substances in rice bran can be effectively converted, and the concentration and activity of the physiologically active substances in fermented products can be significantly improved. In the present study, we introduce a novel method for producing RBE, which utilizes a polar solvent to extract bioactive compounds that would typically be degraded in conventional extraction processes and a fermentation step to improve the bioavailability of the bioactive compounds. Moreover, the RBE is examined *in vitro* and *in vivo* for treating a cosmetic skin condition.

## 2 Materials and methods

### 2.1 Materials

Rice bran was obtained from Synertechcare Australia Pty Ltd. (Australia), the ethanol (analytical grade) was purchased from Sinopharm Chemical Reagent Co., Ltd. (China), and the LC/MS-grade reagents (formic acid, acetonitrile, and methanol) were supplied by Merck KGaA (Germany). Mouse skin fibroblast cells (MSF cells, MZ-2712) were procured from Ningbo Mingzhou Biotechnology Co., Ltd. (China). Dulbecco’s modified Eagle’s medium (DMEM) and fetal bovine serum (FBS) were obtained from Gibco (United States). Streptomycin, 0.25% trypsin–EDTA (1×), and penicillin–streptomycin solution (100×) were obtained from Beyotime Biotechnology (Shanghai, China). Dimethyl sulfoxide (DMSO, 99.7%) was acquired from Sigma-Aldrich GmbH (Steinheim, Germany). The Cell Counting Kit-8 (CCK-8, Cat# K101828133EF5E) was purchased from APExBIO Technology LLC (United States), and the TRIzol reagent was sourced from Thermo Fisher Scientific (United States).

### 2.2 Preparation of RBE

Fragmented plant material derived from a species of the genus *Oryza* was subjected to extraction using 50% v/v ethanol. Ten grams of rice bran were added to 90 mL of the aqueous phase for extraction. The material was incubated with the solvent at 90 °C for 2 h to allow the solubilization of the bioactive constituents. Following extraction, the remaining plant solids were removed by filtration using a 300-mesh filter or centrifugation at 1,000 g to obtain a crude extract. The crude extract was subsequently fermented using a selected microorganism, *Aspergillus oryzae* (ATCC 42149, which was maintained using the general protocol provided by ATCC), at room temperature for a duration of 14 days. The fermentation process aimed to enzymatically degrade macromolecules (e.g., proteins, peptides, and polysaccharides) into smaller bioavailable molecules, such as amino acids and simple sugars. *Aspergillus oryzae* was removed by filtration or centrifugation as described above, followed by inactivation of the solution via heating at 85 °C for 10 min. Volatile organic compounds generated during fermentation were removed from the extract using a benchtop lab freeze dryer (LAB1ST) or similar techniques, yielding the final RBE.

### 2.3 UPLC-QTOF-MS/MS analysis

The experiment required taking 0.5 g of RBE samples and placing them in a 15-mL centrifuge tube, followed by adding 5 mL of 80% methanol and centrifuging at 13,000 rpm for 10 min. The supernatant obtained was passed through a 0.22-μm filter membrane as the experimental sample.

Chromatography separation was implemented using a ZORBAX Extend C18 column (2.1 mm × 100 mm, 1.8 μm, Agilent), with a flow rate of 0.3 mL/min, an injection volume of 1 μL, and a column temperature of 45 °C. The binary mobile phase consisted of 0.1% formic acid in water (A) and acetonitrile (B), and the gradient elution program was: 0 ∼ 0.01 min, 5% B; 0.01 ∼ 9.00 min, 5% ∼ 82% B; 9.00–20.00 min, 82% ∼ 95% B; 20.00–26.00 min, 95% B; and 26.00 ∼ 28.00 min, 95% ∼ 5% B.

An electrospray ion source (ESI) was used, and scanning was conducted in the positive and negative ion modes. The MS conditions were as follows: scan range, 100–1,500 m/z (MS) and 50–1,000 m/z (MS/MS); curtain gas, 35 psi; ion spray voltage floating, −4,500 V or 5,000 V; ion source temperature, 500 °C; declustering potential, −60 V or 60 V. To obtain the fragmentation information of the metabolites, the MS/MS mode was used, nitrogen was used as the collision gas, and the collision energy was run at an alternating voltage from −40 to 40 eV (low energy to high energy). The collision energy spread was 10 eV.

### 2.4 *In vitro* human dermal fibroblast cell culture

#### 2.4.1 *In vitro* RBE treatment

Mouse skin fibroblasts (MSF, passage 5) were cultured in DMEM supplemented with 10% (v/v) FBS, 2 mML glutamine, 100 U/mL penicillin, and 100 μg/mL streptomycin. In each 96-well, MSF was drop-seeded at a density of 3.0 ×10^4^ cells/mL and allowed to attach on the surface for 24 h before adding RBE. RBE at the concentrations of 0.01, 0.1, 1, 10, and 100 μg/mL was then added to each well in triplicate, and all cells were incubated in a static cell culture system at 37 °C and 5% CO_2_. Cell proliferation was measured using a CCK8 assay kit (K101828133EF5E) at 24 and 48 h.

#### 2.4.2 *In vitro* cell scratch assay

MSF migration was further examined using a wound scratch assay. Briefly, cells at a density of 3 × 10^5^ cells/mL were plated in 6-well plates. After 24 h, 200-μL pipette tips were used to scratch the cell layer. After washing with PBS twice to remove cell debris, serum-free medium containing different concentrations of RBE was added to continue culturing. At 12, 24, 36, and 48 h, microscopic images were taken with a ×4 objective prior to assessment using ImageJ. All the experiments were performed in triplicate.

#### 2.4.3 Preparation of 3D MSF cell spheroids

MSF cells at a density of 5.0 × 10^4^ cells/mL were seeded in 100 μL of cell culture medium per well in ultra-low attachment (ULA) 96-well plates (round bottom, Costar) (Bresciani et al., 2019; Sherman et al., 2018). The plates were then centrifuged at 1,000 rpm for 5 min to ensure immediate close contact of cells. After 48 h, the cells formed a compact spherical structure, and the cell spheres were maintained by the semi-liquid exchange method. The liquid exchange interval was 48 h.

#### 2.4.4 *In vitro* 3D MSF cell spheroids treated with RBE

To determine the effect of RBE on three-dimensional (3D) MSF cell spheroids, the 3D cell spheroids were incubated with RBE at a concentration of 1 μg/mL. The mRNA expression of COLI and COL III was evaluated by comparing 3D cell spheres with 2D cells treated with the same concentration of REB and control group without REB treatment. The response to 3D models was compared to non-treatment, assessing for mRNA expressions of COL I and COL III.

#### 2.4.5 Construction of a three-dimensional skin model

MSF cells were combined with rat tail collagen (Sigma, C3867) to form a 3D structure on a Transwell insert, which was placed in a 24-well plate. This structure is referred to as the dermal layer of the skin. Once the MSF layer solidified, mouse keratinocytes were combined with rat tail collagen to form the epidermal layer.

After the complete 3D structure was formed, cell culture media were added to the bottom chamber of the insert to provide nutrients to the cells. At 48 h post-seeding, RBE (20 μL of 1 μg/mL) was added to the top of the epidermal layer, just enough to cover the surface. The setup was then incubated for 3 days, and the entire 3D construct on the Transwell membrane was carefully cut off and fixed with 4% paraformaldehyde for 48 h. The fixed samples were dehydrated, transparentized, and paraffin-embedded by standard procedures previously reported (Fang et al., 2025; Hu et al., 2024). The slice thickness was 5 μm, followed by standard hematoxylin–eosin (HE) staining.

### 2.5 *In vivo* assessment of RBE in mouse skin

Male, pathogen-free, Balb/c mice at 12 weeks of age (weighing 24.8 ± 0.6 g) were used to investigate the effect of RBN on the skin. All animals were housed in cages with free access to water and food. The environment was controlled at 24 °C–26 °C and 44%–46% humidity under a 12:12-h light–dark cycle with lights on at 6 a.m. All protocols were approved by the Animal Ethics Committee at Nanjing University of Chinese Medicine (Approval Number: 202302A012). Briefly, the dorsal area was shaved, followed by division into two equal areas. Cream containing RBE was added to one area, whereas the other equal area of skin received blank cream only and was used as the non-treatment group. All the treatment procedures were continued daily for a period of 14 days and 28 days. On each termination date, the animals were euthanized and skin samples were collected for histological and molecular analyses.

### 2.6 Histology and immunofluorescent staining

Mouse skin tissue collected on days 14 and 28 post-RBE treatment was harvested and fixed in 10% (v/v) formalin for 24 h. Thereafter, tissues were processed and embedded in paraffin and sectioned at a thickness of 5 μm prior to staining with hematoxylin and eosin (H&E) for the analysis of skin structure. Sections of the tissue samples from days 14 and 28 were also stained with Mason’s trichrome for the analysis of the percentage of positively stained fibers using ImageJ. Elastin expression in the skin before and post-RBE treatment was analyzed using primary antibodies (anti-elastin 1:200, Abcam, anti-elastin antibody (ab21610)). The expression of elastin IFC staining was assessed by two independent researchers blinded to the identity of the samples using ImageJ.

### 2.7 Skin mechanical property test

The skin mechanical property test was conducted using a tensile testing machine (TH-8203A). Fresh skin tissue was harvested and collected from both the treatment side and the non-treatment side of each mouse. All samples were then cut into 20 mm × 50 mm strips and mounted between the grips of the machine. The elasticity test was performed at a speed of 50.0 mm/min. Stress–strain curves were produced by calculating the force applied to each sample and the displacement of the sample itself. The data are represented as Young’s modulus and tensile strength.

### 2.8 Skin elasticity test

**FIGURE 1:**
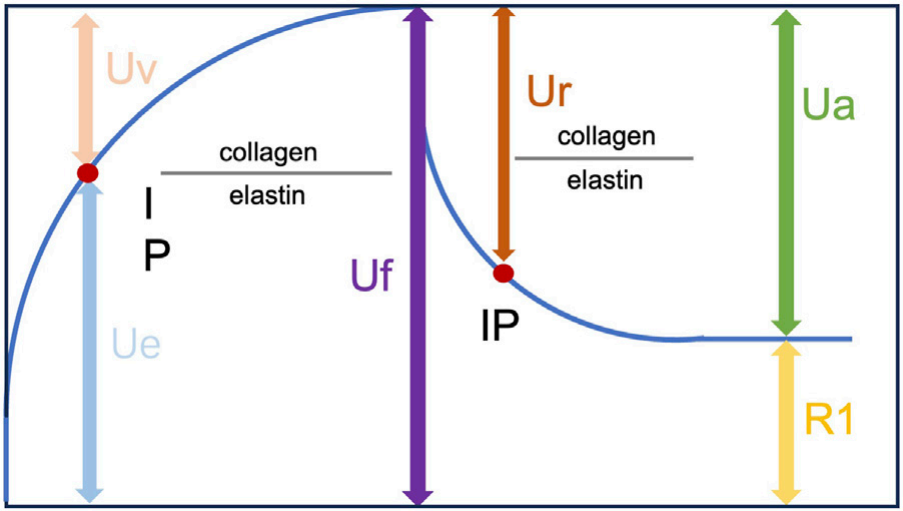
Skin deformation curve obtained with a Cutometer.

Skin elasticity was evaluated with a Cutometer^®^ (Courage & Khazaka Co., Germany), which is an instrument to assess the skin's elastic properties using suction and elongation. On days 14 and 28, the animals were anesthetized with isoflurane (3%), and the skin was carefully cleaned with a cotton swab soaked in warm distilled water. The cutometer probe was attached to the skin of the area to be tested, allowing the test area to be sucked into the probe aperture. Measurements were repeated three times (suction time: 2 s; relaxation time: 2 s). The evaluated parameters were R2 (Ua/Uf), which is the gross elasticity of the skin and includes viscous deformation, and R7 (Ur/Uf), which is the biological elasticity, and is the ratio of the immediate retraction to the total distension (Figure 1) (Krueger et al., 2011).

### 2.9 Transepidermal water loss (TEWL) determination

Skin water-loss post-RBE treatment was examined using TEWL (Levin and Maibach, 2005). On days 14 and 28, the animals were anesthetized with isoflurane (3%), and their skin was carefully cleaned with a cotton swab soaked in warm distilled water. An open-chamber Tewameter^®^ TM Hex (Courage & Khazaka Co., Germany) was used to record the TEWL on each side of the dorsal area (treatment vs. non-treatment), with the value expressed in g/m^2^/h. Measurements were performed for 120 s after the application of the detecting probe to the skin. Three independent measurements of the same skin area were averaged for each value.

### 2.10 RNA isolation and quantitative real-time polymerase chain reaction

Skin tissues were collected from mice on days 14 and 28 post-RBN treatment from both the treated and control groups. The tissues were used for RNA extraction using the TRIzol (Invitrogen, Carlsbad, CA, United States) method. Total mRNA (1 μg) was reverse-transcribed into complementary DNA using the SensiFAST cDNA synthesis kit (Bioline, London, United Kingdom). Real-time PCR was then carried out using the SsoAdvancedTM Universal SYBR Green Supermix kit (Bio-Rad, Hercules, CA, USA) on the CFX ConnectTM real-time PCR detection system (Bio-Rad) according to the manufacturer’s instructions. Table 1 displays the sequences of primers used for qRT-PCR.

**TABLE 1:** Primer sequence (Sangon Biotech, Shanghai, China).

Gene	Primer	Sequence (5′–3′)
β-actin	Forward	GGC​TGT​ATT​CCC​CTC​CAT​CG
	Reverse	CCA​GTT​GGT​AAC​AAT​GCC​ATG​T
Col I	Forward	AGC​ACG​TCT​GGT​TTG​GAG​AG
	Reverse	GAC​ATT​AGG​CGC​AGG​AAG​GT
Col III	Forward	ACG​TAA​GCA​CTG​GTG​GAC​AG
	Reverse	CAG​GAG​GGC​CAT​AGC​TGA​AC

### 2.11 Statistical analysis

GraphPad Prism 10 software (GraphPad Software Company, San Diego, CA, United States) was utilized for statistical analysis. Statistical significance was assessed using both one-way and two-way ANOVA, with the results expressed as means ± standard deviation. A p-value less than 0.05 was considered statistically significant.

## 3 Results

### 3.1 UPLC-Q-TOF-MS/MS profiling of RBE

A qualitative analysis of RBE was conducted using UPLC-QTOF-MS/MS. We identified eight chemical compounds in RBE, according to the information on ions, secondary fragment ions, reference substances, and literature. The retention times and spectrometric data of RBE are shown in Table 2. Its chemical profile included lipids and polyphenols. The identified lipids were γ-tocotrienols, δ-tocotrienols, and squalene.

**TABLE 2:** Chemical constituents identified in RBE using UPLC-Q-TOF-MS.

Peak No.	Phytochemical Compound	Rt (min)	Molecular Formula	Calc. MW	Error (ppm)	Ms^1^ (m/z)	Ms^2^ (m/z)
1	Azelaic acid	6.896	C_9_H_16_O_4_	188.1094	0.09	187.0098 [M-H^+^]^-^	57.0348, 97.0658, 169.0875, 143.1076, and 125.0978
2	Ferulic acid	7.132	C_10_H_10_O_4_	193.0526	0.16	236.0932 [M + CH_3_CN-H]^-^	134.0372, 149.0634, and 178.0278
3	Pinellic acid	8.817	C_18_H_34_O_5_	330.2406	0.06	329.2366 [M-H^+^]^-^	99.0832, 127.1139, 139.1145, 171.1050, 183.1411, 193.1251, 211.1368, 229.1475, 293.2144, and 311.2245
4	9,10,13-Trihydroxy-11-octadecenoic acid	9.287	C_18_H_34_O_5_	330.2406	0.06	329.2364 [M-H^+^]^-^	99.0819, 129.0932, 139.1142, 155.1093, 171.1040, 199.1357, 201.1156, 211.1359, 229.1466, 275.2039, 293.2142, and 311.2253
5	γ-Tocotrienols	11.093	C_28_H_42_O_2_	410.3185	3.91	415.3217 [M-H^+^]^-^	115.9221, 132.9243, 205.1617, 295.2309, 311.1710, and 381.1149
6	δ- Tocotrienols	11.716	C_27_H_40_O_2_	396.3028	−0.25	436.0693 [M + CH_3_CN-H]^-^	115.9218 and 311.2045
7	Dihydroferulic acid dimer	13.826	C_20_H_28_O_8_	380.1838	0.05	379.2370 [M-H^+^]^-^	116.9290 and 333.2309
8	Squalene	21.237	C_30_H_50_	410.3910	−0.05	491.3457 [M+2xCH_3_CN-H]^-^	99.9264, 115.9920, 149.1309, 200.8063, 222.8442, 273.2603, 341.3357

### 3.2 Examination of the effect of RBE on skin cell proliferation and migration *in vitro*

**FIGURE 2:**
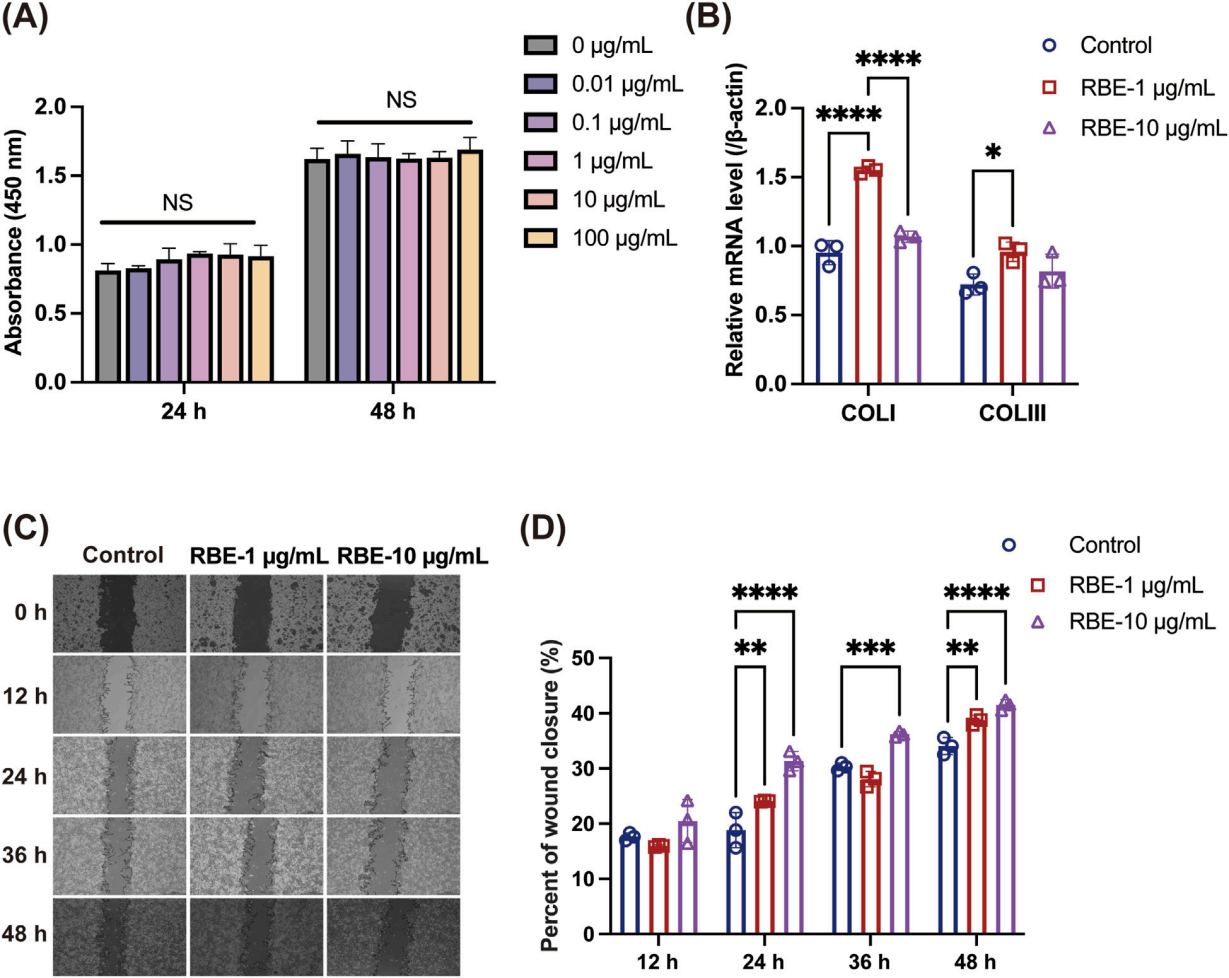
Evaluation of the *in vitro* activity of RBE. **(A)** Effect of RBE on the viability of MSF cells. **(B)** qRT-PCR was used to evaluate the effect of MSF collagen gene expression at 24 h after treatment with RBE, and qRT-PCR outcomes are shown relative to β-actin. **(C,D)** Representative images of the scratch wound healing assay at 0, 12, 24, 36, and 48 h, along with the percentage of wound closure determined by the microscopic photographs. All data represent the mean ± standard deviation (n = 3, *p < 0.05, **p < 0.01, ***p < 0.005, and ****p < 0.001).

RBE has been reported to have antioxidant activity in previous studies (Andriani et al., 2022); therefore, we examined the effect of RBE at different concentrations ranging from 0.01 to 100 μg/mL on the proliferation of mouse skin fibroblasts (MSFs). The results of the cell viability assay showed that all concentrations of RBE promoted the proliferation of MSF cells to a certain extent, but there was no significant difference compared to the control group (Figure 2A). At the gene expression level, the expression levels of type I and type III collagen mRNA were significantly upregulated after 1 μg/mL RBE treatment for 24 h (Figure 2B), suggesting its potential to promote extracellular matrix synthesis. In order to further explore the effect of RBE on cell migration behavior, we selected two concentrations of 1 μg/mL and 10 μg/mL for the scratch test. Quantitative analysis showed that the 10 μg/mL RBE-treated group achieved wound closure rates of approximately 31.36% ± 1.75%, 36.23% ± 0.51%, and 41.53% ± 0.94% at 24, 36, and 48 h (****p < 0.001) (Figures 2C, D), respectively, which were significantly different from those of the control group. Based on the above results, 1 μg/mL RBE showed good activity in promoting collagen gene expression and cell migration, so it was determined as the optimal concentration for subsequent experiments.

### 3.3 RBE promotes mRNA expression of collagen I and III in 3D cell spheroids of MSF

**FIGURE 3:**
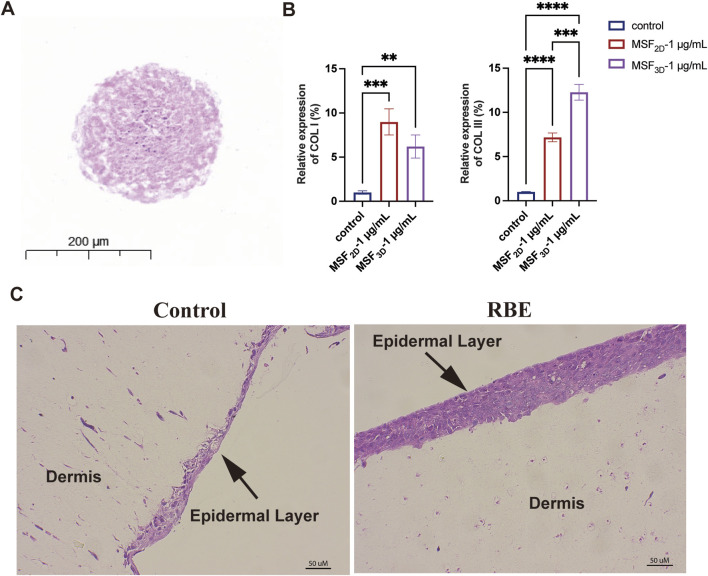
Evaluation of the activity of MSF_3D_ spheroids by RBE. **(A)** Hematoxylin and eosin (H&E) staining of MSF_3D_ spheroids. **(B)** qRT-PCR was used to evaluate the effect of MSF_3D_ spheroids on collagen gene expression at 48 h after treatment with RBE. **(C)** H&E staining of the 3D skin model treated with RBE (n = 3, **p < 0.01, ***p < 0.005, and ****p < 0.001).

We next employed a 3D spheroid model of MSF to further investigate the effect of RBE on the mRNA expression of collagen types I and III (COL I and COL III). As shown in Figure 3A, MSF 3D spheroids were successfully fabricated, as previously described (Shi et al., 2022). After treatment with RBE (1 μg/mL) in both 2D-cultured MSF and 3D MSF spheroids, the mRNA level of COL I, the most abundant collagen type, was significantly upregulated by 8.99-fold in 2D and 6.20-fold in 3D (Figure 3B). Interestingly, the expression of the immature collagen type III was also significantly increased, with a more pronounced effect observed in the 3D MSF spheroids, showing a 12.28-fold increase compared to a 7.17-fold increase in the 2D model (Figure 3B). These results suggest that RBE stimulates greater expression of collagen III in 3D MSF spheroids, which are better at mimicking the environment of skin and other soft tissues. Additionally, we established a 3D *ex vivo* skin model composed of both MSFs and mouse keratinocytes to further assess the effects of RBE (Figure 3C). H&E staining also revealed a healthier epidermal layer and improved overall skin architecture following RBE treatment.

### 3.4 RBE treatment accelerated elastin regeneration in skin

**FIGURE 4:**
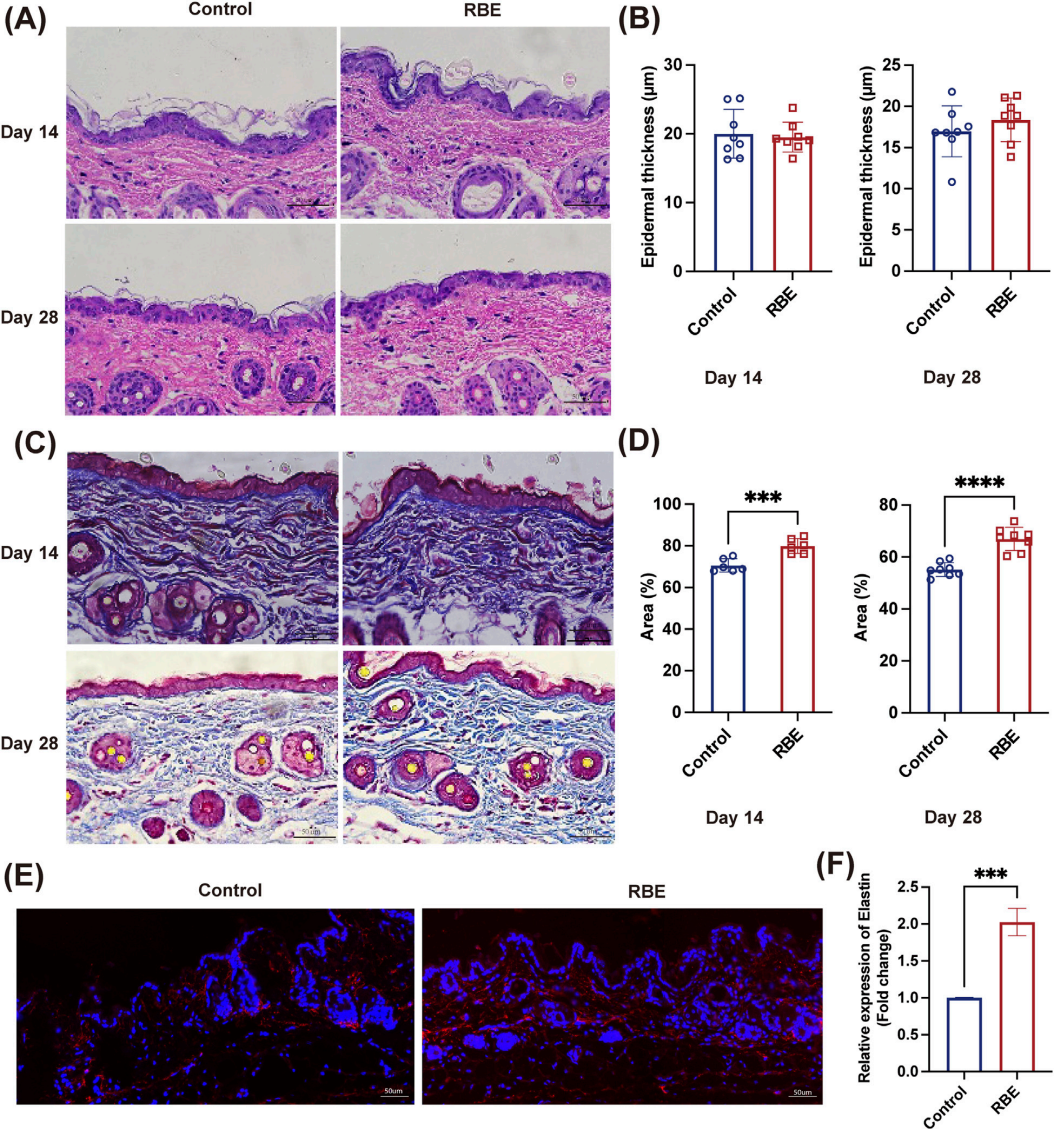
**(A,B)** Mouse skin was stained with H&E; the thickness of the epidermis was statistically significant. **(C,D)** Mouse skin was stained with Masson’s trichrome staining, and collagen deposition was statistically significant. **(E,F)** Mouse skin was immunofluorescence-stained, and the expression of elastin was found to be statistically significant. All data represent the mean ± standard deviation (n = 3, ***p < 0.005, and ***p < 0.001).

RBE was then applied to mouse skin for 14 days and 28 days to evaluate its effect on skin characteristics (Figure 4). H&E staining and analysis showed a comparable skin structure before and post-RBE treatment on days 14 and 28 (Figure 4A). Epidermal thickness remained constant at approximately 18.71 ± 3.00 μm, suggesting that RBE had minimal effects on keratinocyte and epidermal formation (Figure 4B). Additionally, we also assessed collagen formation in the skin by Masson’s trichrome staining (Figure 4C). The percentage of collagen was significantly higher in the RBE-treated skin group than in the untreated control groups on days 14 and 28 (Figure 4D). The percentage of collagen increased from 70.61% ± 3.14% to 79.95% ± 3.56% after 14 days of treatment, while enhanced collagen formation was more notable on day 28 post-treatment, with a recorded 66.96% ± 4.47% for the RBE-treated group versus 55.16% ± 2.69% for the control group. These data confirm that RBE treatment accelerates collagen regeneration in the skin, which is in agreement with previous literature on rice extract (Linsaenkart et al., 2023). After 28 days of RBE treatment, the skin was further processed for immunofluorescent analysis of elastin (Figure 4E). As shown in the images, more elastin fibers stained in red were observed in the treated skin. Quantitative analysis revealed a significant regeneration of elastin fibers at approximately 2.03 ± 0.11 fold post-RBE treatment (Figure 4F). All these data prove that RBE induces skin collagen and elastin regeneration, presenting potential for anti-aging skin treatment.

### 3.5 RBE treatment enhanced skin elasticity

**FIGURE 5:**
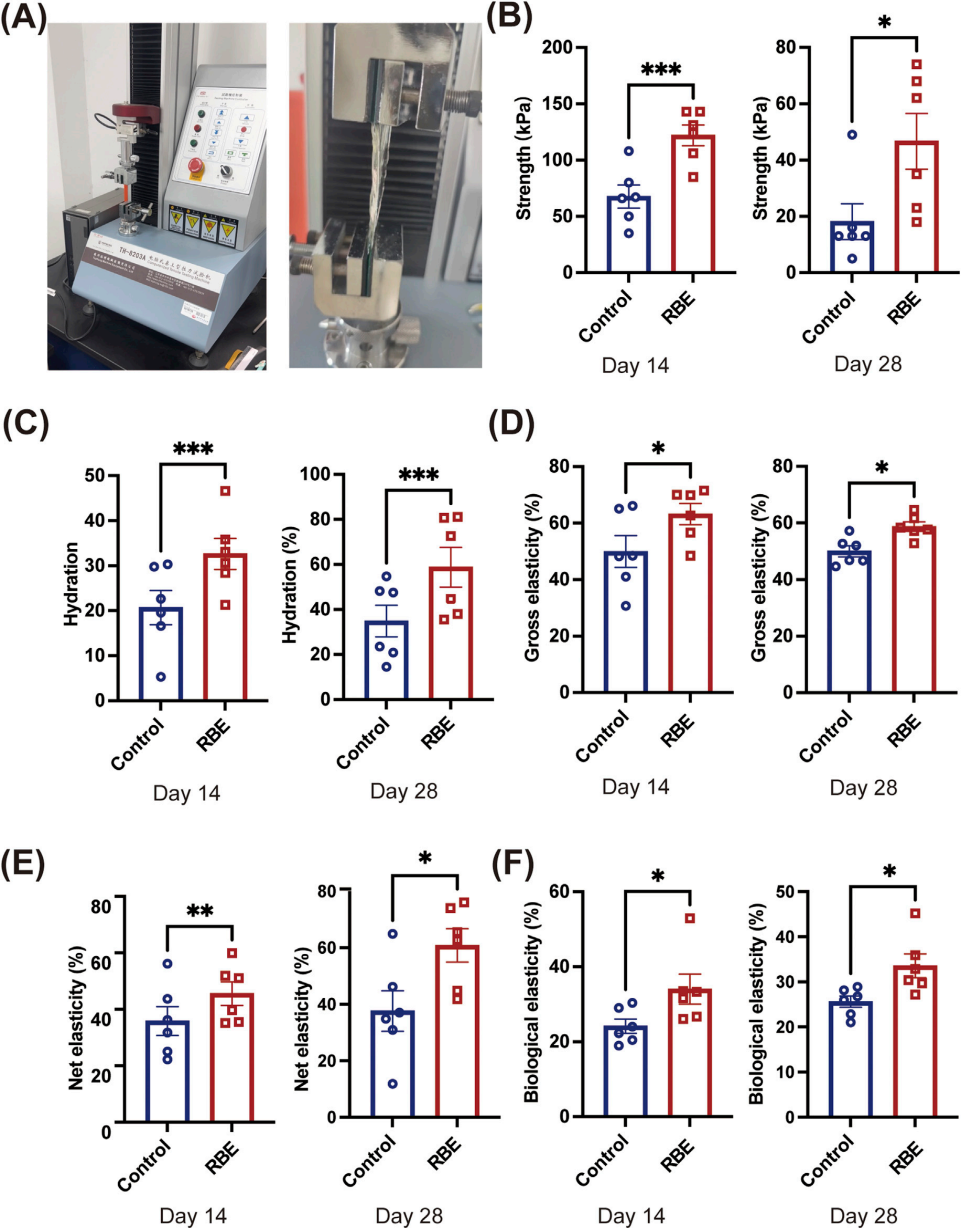
Effects of rice bran extract on the skin condition of Balb/c mice. **(A)** Schematic diagram of skin tension detection; **(B)** Test results of skin tensile strength; **(C)** Test results of skin hydration; **(D)** Test results of skin cross elasticity; **(E)** Test results of skin net elasticity; **(F)** Test results of skin biological elasticity. All data represent the mean ± standard deviation (n = 6, *p < 0.05, **p < 0.01, and ***p < 0.005).

The mechanical properties of the skin on days 14 and 28 post-RBE treatment were further evaluated using a TH-8203A Mechanical Tester (Figure 5A). Stress–strain curves were plotted based on the stress needed to break the skin and measured as tensile strength and the strain on the skin when it broke (elongation to the breaking point). Skin samples after 14 days of treatment with RBE had significantly increased tensile strength, which was approximately 2 times higher than that of the control skin (Figure 5B). Moreover, skin strength was further enhanced after 28 days of treatment to approximately 18.17 ± 15.55 kPa for treated skin and 46.67 ± 24.31 kPa for the untreated control skin (Figure 5B), indicating a greater ability to absorb more energy before breaking. Moreover, a similar tendency was noted in the hydration measurement. At both time points of RBE treatment, significantly higher hydration was recorded than that for the untreated control skin (Figure 5C). We then evaluated the cross elasticity, net elasticity, and biological elasticity of the skin treated with RBE. Cross elasticity was measured at 49.93 ± 13.76% for normal mouse skin, which increased to 63.19 ± 9.18% and 58.73 ± 4.07% after 14 and 28 days of treatment, respectively (Figure 5D). Net elasticity was also found to be significantly increased post-RBE treatment (Figure 5E). Net elasticity reached 45.54% ± 10.19% and 60.79% ± 14.50% on days 14 and 28, respectively, compared to 35.84% ± 12.61% and 37.61% ± 17.52% in normal skin at the same time-points. Additionally, biological elasticity increased from 24.18% ± 4.61% in normal skin to 34.03% ± 9.80% in the RBE-treated skin on day 14, and a similar tendency was also recorded after 28 days of RBE treatment (Figure 5F). These data indicate that skin tissue that underwent RBE treatment was significantly stronger and more elastic than that in normal mice.

## 4 Discussion

In this study, we comprehensively investigated the bioactive profile and skin-regenerative potential of RBE using a combination of chemical profiling, *in vitro* assays, 3D skin models, and *in vivo* analyses. Our findings collectively demonstrate that RBE contains a rich mixture of bioactive compounds with promising regenerative effects on the skin, particularly by promoting collagen and elastin synthesis in the skin, enhancing fibroblast migration, and improving skin mechanical properties.

UPLC-QTOF-MS/MS profiling identified eight key compounds in RBE, primarily lipids and polyphenols, including ferulic acid, γ-tocotrienols, δ-tocotrienols, and squalene. These compounds are known for their antioxidant and anti-inflammatory properties (Zaaboul and Liu, 2022), which may underlie the observed bioactivities on skin cells. Tocotrienol can protect the skin from inflammation, ultraviolet radiation, and melanin accumulation, and it can effectively prevent skin aging (Ghazali et al., 2022). Ferulic acid has anti-inflammatory, antioxidant, antibacterial, and other activities, which can protect fibroblasts and accelerate angiogenesis and wound healing, and have been widely used in skincare formulations (Zduńska et al., 2018). The presence of these lipophilic antioxidants supports the fact that RBE has potential in protecting and regenerating skin tissue, which is consistent with prior reports on rice-derived extracts (Wang Q. et al., 2025). *In vitro* experiments revealed that RBE at concentrations of 1 μg/mL and 10 μg/mL significantly enhanced MSF proliferation and migration. Interestingly, while 1 μg/mL of RBE promoted both proliferation and collagen gene expression, 10 μg/mL primarily enhanced cell migration, suggesting a concentration-dependent bifunctionality.

In addition, in order to more realistically simulate the interaction between the microenvironment and cells *in vivo*, a 3D cell sphere model was established to evaluate the activity of RBE. In 2D culture and 3D MSF spheroids, RBE treatment significantly up-regulated the expression of type I collagen in MSF cells and the expression of type III collagen in MSF 3D spheroids (approximately 12-fold). Collagen III, which plays a key role in early wound healing and is abundant in pliable tissues such as the skin (Yan et al., 2025), was particularly responsive to RBE in the 3D model. This indicates that the 3D spheroid environment more accurately mimics *in vivo* skin conditions and further validates RBE’s role in supporting extracellular matrix (ECM) synthesis. The 3D *ex vivo* skin model also demonstrated improved epidermal structure after RBE application, confirming its tissue-level benefits. In animal experiments, RBE was applied for more than 14 days and 28 days. The results of tissue staining showed that the content of collagen in the skin after RBE treatment increased by 10% compared with the control group. The results of immunofluorescence staining confirmed that RBE could increase the content of elastin in the skin. These results are in agreement with the *in vitro* results, confirming the great potential of RBE utilization in dermatology and wound healing. Masson’s trichrome staining showed a notable increase in collagen deposition, while elastin fiber regeneration was markedly enhanced after 28 days. Taken together, these outcomes align with prior literature on rice bran derivatives and further substantiate the skin-rejuvenating effects of RBE.

Importantly, mechanical analysis of skin tissues post-treatment revealed significantly improved skin tensile strength, hydration, and elasticity, including cross, net, and biological elasticity. These biomechanical enhancements suggest that RBE not only supports ECM regeneration but also contributes to restoring the functional integrity of aging or damaged skin (Martyts et al., 2024). In summary, our data here provide strong evidence that RBE exerts multifaceted effects on skin health, ranging from molecular stimulation of collagen and elastin synthesis to functional improvement in elasticity and hydration. These findings position RBE as a promising candidate for topical formulations aimed at skin regeneration, wound healing, and anti-aging therapies.

## 5 Conclusion

In this study, we investigated the protective effects of RBE on the skin. The RBE was found to significantly increase the synthesis of collagen and elastin in the skin, elevate the skin moisture content, and enhance the migration of skin fibroblasts. These findings indicate that RBE has significant benefits for maintaining and improving human skin health. Future research can delve deeper into its mechanism of action, optimize the preparation method, and conduct more clinical trials to fully assess its potential in maintaining skin health and combating skin aging.

## Data Availability

The original contributions presented in the study are included in the article/supplementary material; further inquiries can be directed to the corresponding authors.
